# Hepatitis E Virus (HEV) Spreads from Pigs and Sheep in Mongolia

**DOI:** 10.3390/ani13050891

**Published:** 2023-03-01

**Authors:** Enkhbaatar Batmagnai, Bazartseren Boldbaatar, Amarbayasgalan Sodbayasgalan, Yuko Kato-Mori, Katsuro Hagiwara

**Affiliations:** 1 Laboratory of Virology, Institute of Veterinary Medicine, Mongolian University of Life Sciences, Ulaanbaatar 17024, Mongolia; 2 Department of Infectious Diseases and Microbiology, School of Veterinary Medicine, Mongolian University of Life Sciences, Ulaanbaatar 17024, Mongolia; 3 Graduate School of Science, Technology and Innovation, Kobe University, 7-1-49 Minatojima Minami-Machi, Chuo-ku, Kobe 650-0047, Japan; 4 Department of Pathobiology, School of Veterinary Medicine, Rakuno Gakuen University, Hokkaido 069-8501, Japan

**Keywords:** hepatitis E, pig, sheep, prevalence, Mongolia, phylogenetic analysis

## Abstract

**Simple Summary:**

Hepatitis E virus (HEV) is a zoonotic pathogen, with an increasing number of cases worldwide. In Asia, including Mongolia, infections are associated with the zoonotic HEV-3 and HEV-4 genotypes, and pigs, deer, and wild boars are the main reservoirs. Recent studies have revealed that sheep are hosts of the virus in several countries. The aim of our study is to diagnose HEV RNA in feces and liver samples of sheep in Mongolia and clarify the origin of the virus and characterize its chain of infection. From our results, we found HEV genotype 4 in sheep and it was closely related to pig HEV genotype 4 in the same region. On Mongolian pig farms, pigs are fed with the raw internal organs of sheep for fattening the pigs as a free resource of protein. There is a concern that the spread of HEV could affect livestock feeding.

**Abstract:**

Hepatitis E is a viral infectious disease in pigs, wild boars, cows, deer, rabbits, camels, and humans as hosts caused by *Paslahepevirus*. Recently, it has been detected in a wide variety of animals including domestic small ruminants. Mongolia is a land of nomadic people living with livestock such as sheep, goats, and cattle. Due to how Mongolian lifestyles have changed, pork has become popular and swine diseases have emerged. Among them, Hepatitis E disease has become a zoonotic infectious disease that needs to be addressed. The HEV problem in pigs is that infected pigs excrete the virus without showing clinical symptoms and it spreads into the environment. We attempted to detect HEV RNA in sheep which had been raised in Mongolia for a long time, and those animals living together with pigs in the same region currently. We also conducted a longitudinal analysis of HEV infection in pigs in the same area and found that they were infected with HEV of the same genotype and cluster. In this study, we examined 400 feces and 120 livers (pigs and sheep) by RT-PCR in Töv Province, Mongolia. HEV detection in fecal samples was 2% (4/200) in sheep and 15% (30/200) in pigs. The results of ORF2 sequence analysis of the HEV RT-PCR-positive pigs and sheep confirmed genotype 4 in both animals. The results suggest that HEV infection is widespread in both pigs and sheep and that urgent measures to prevent infection are needed. This case study points to the changing nature of infectious diseases associated with livestock farming. It will be necessary to reconsider livestock husbandry and public health issues based on these cases.

## 1. Introduction

The hepatitis E virus (family *Hepeviridae*; subfamily *Orthohepevirinae*; genus *Paslahepevirus*) is one of the main viral causes of acute human hepatitis worldwide [1]. Out of eight different genotypes, HEV-3 and HEV-4 are confirmed as zoonotic. Suidae are generally recognized as the main reservoirs of these genotypes. HEV-4 has been identified in China in goats [2], cows [3], cow’s milk [4], and sheep [5]. The disease is endemic in Asia, Africa, and Latin America [6]. 

The presence of anti-HEV antibodies in sheep and goats has been reported worldwide [7,8]. High similarity between human and ruminant HEV sequences has also been confirmed [9,10]. This raises concerns about the zoonotic transmission of HEV from these animal species [10,11]. In connection with this, it has been suggested that the consumption of contaminated milk, meat, and/or dairy products from sheep could be a source of HEV infection in humans [12,13]. However, information about the relationship between other livestock and sheep is not well understood. 

Mongolia is a country of animal husbandry with 30 million sheep [14]. It also has the third-largest population of sheep in the world [15]. Sheep meat is considered a staple food for Mongolians. Due to recent dietary changes, pig farming is being promoted in Mongolia. Most of the pig farms are located in Töv Province. Currently, ham, bacon, liver paste, and sausages are common foods for Mongolians that are prepared from pork at pig farms in Töv Province. This situation must be considered an important public health issue for food safety. There have been several studies on the molecular characterization of HEV in both pigs and human patients in Mongolia [16,17,18,19,20]. In a report on Mongolians, HEV prevalence was 12%, which is higher than in Russia (1.2%) and Japan (3%), despite lower pork consumption in Mongolia compared to the other countries. Although there have been reports of HEV infection in sheep from China, there is no information regarding HEV transmission from sheep in Mongolia. The purpose of this study was to determine the risk of HEV infection in native sheep associated with the introduction of pigs into livestock in Mongolia.

## 2. Materials and Methods

### 2.1. Samplings

**Figure 1:**
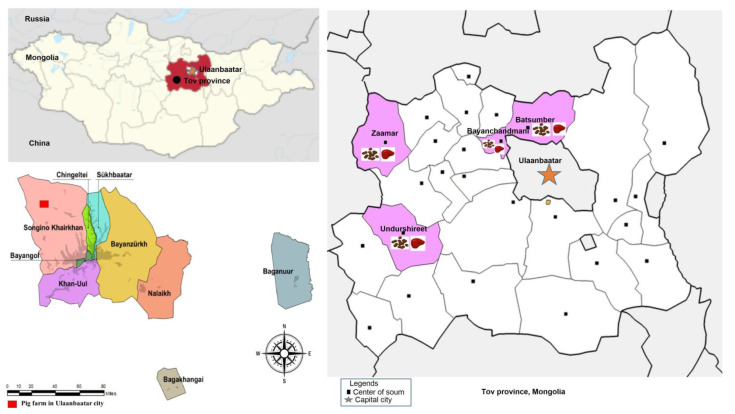
Sampling locations in Mongolia. The **upper left** figure shows the whole Mongolian territory with two neighboring countries and the locations of Töv Province and the capital city, Ulaanbaatar. The **lower left** figure shows the whole territory of Ulaanbaatar city and the location of the pig farm in Songino Khairkhan district in the red rectangle. The figure on the **right** shows the whole territory of Töv Province and the locations of four soums are shown in pink (Zaamar, Undurshireet, Bayanchandmani, and Batsumber soums) where we collected the feces and liver samples of the sheep. In addition, the figure shows that the Bayanchandmani and Batsumber soums are near to the pig farm located in Songino Khairkhan district. Emeelt—the main slaughterhouse in Mongolia—is also in Songino Khairkhan district. The red rectangles show the areas where the pig feces samples were collected.

This was a cross-sectional study conducted in Töv Province and Ulaanbaatar city (Figure 1) between 2020 and 2022. Considering a 95% confidence interval (95% CI) and the desired precision of ±5%, the sample size was calculated as 384. We sampled 400 feces (200 sheep feces and 200 pig feces), and 120 livers (60 sheep livers and 60 pig livers). Sheep fecal and liver samples were equally collected in the Bayanchandmani, Batsumber, Undurshireet, and Zaamar soums (districts) in Töv Province. The liver and fecal samples were transported to the laboratory on ice. The Bayanchandmani and Batsumber soums are close to Emeelt slaughterhouse, which is the main slaughterhouse in the central region of Mongolia. The study area for the sheep was set near Ulaanbaatar city, where pig farming is most prevalent in Mongolia. Sheep and free-roaming livestock were raised in the same region as the pig farms. The fecal samples were collected from 200 pigs and the liver samples were collected from 60 pigs slaughtered in Ulaanbaatar city. The ORF2 region of HEV RNA was a target sequence for viral detection [21]. In addition, the farmers were asked questions that included general knowledge about HEVs and possible contact between livestock to analyze risk factors.

### 2.2. RNA Extraction and Detection of HEV RNA by RT-PCR

Fecal samples from sheep and pigs were suspended 10% (w.p.v.) in sterile PBS. The fecal and liver samples were homogenized with zirconium beads in a tissue lyser (QIAGEN, Hilden, Germany). Next, the homogenized samples were centrifuged at 8000 rpm for 5 min, and the supernatants were collected in sterile tubes. RNA from pig livers, experimentally infected with HEV, was used as a positive control, and non-HEV-infected healthy pigs were used as a negative control [22]. Viral RNA was extracted from fecal supernatants using a QIAamp Viral RNA extraction kit (QIAGEN, Hilden, Germany) according to the kit’s protocol, and 50 mg from each liver tissue was lysed with 1 mL Trizol (Invitrogen, Carlsbad, CA, USA). RNA was precipitated with 0.5 mL isopropanol and washed with 1 mL of 75% ethanol. The RNA was solubilized in 20 µL RNase-free water [21]. A one-step RT-PCR kit (QIAGEN, Hilden, Germany) was used to amplify the ORF2 region of the HEV (primer sequences used: ORF2-F1; (5772-5794) 5′-AATTATGCYCAGTAYCGRGTTG-3′, ORF2-R1; (6416-6439) 5′-CCCTTRTCYTGCTGMGCATTCTC-3′). The reaction conditions were reverse transcription at 45 °C for 60 min and 95 °C for 15 min as a PCR cycle, denaturation at 94 °C for 30 s, annealing at 55 °C for 30 s, extension at 72 °C for 75 s for 35 cycles, and final extension at 72 °C for 7 min [21]. Nested PCR: the RT-PCR products were then diluted and amplified again using Takara Ex-Taq (Takara Bio. Inc., Shiga, Japan) to check the PCR amplification of the F1-R1 region (primer sequences used [23]: ORF2-F2 (5953-5975) 5′-GTWATGCTYTGCATWCATGGCT-3′, ORF2-R2 (6341-6363) 5′-AGCCGACGAAATCAATTCTGTC-3′) under the following conditions: 95 °C for 2 min (1 cycle); 94 °C for 30 s, 55 °C for 30 s, 72 °C for 30 s (35 cycles); and 72 °C for 5 min (1 cycle). The nested PCR products were electrophoresed on a 1.5% agarose gel, and amplified RNA bands were detected using a transilluminator (Toyobo-FAS-III, Toyobo Co., Ltd., Osaka, Japan) followed by ethidium bromide staining. The expected amplified HEV RNA band (348 bp) was sequenced after purification [21].

### 2.3. DNA Sequencing

The PCR products from each DNA sample were purified using a FastGene Gel/PCR Extraction Kit (NIPPON Genetics Co., Ltd., Tokyo, Japan) and sequenced using an ABI Prism Big Dye Terminator v3.1 Cycle Sequencing Kit (Applied Biosciences, Foster City, CA, USA). The sequences were analyzed using a 3500 Genetic Analyzer (Life Technologies, Carlsbad, CA, USA) [21]. 

### 2.4. Phylogenetic Analysis

The ORF2 gene sequences were then compared with those in the NCBI GenBank database using the multiple alignment function of the online ClustalW tool. Phylogenetic trees were constructed using the neighbor-joining method in MEGA (version X). A total of 28 sequences were submitted to GenBank through an online submission system and accession numbers were obtained and compared with previously reported sequences [24]. The same data were utilized to generate an ML phylogenetic tree, which was initially used to conduct the Bayesian maximum clade credibility (MCC) host a discrete traits tree by using the software BEAST v1.8.4 (http://tree.bio.ed.ac.uk/software/beast/, accessed on 12 June 2022). The strict clock and the best fit GTR + G + I nucleotide substitution model with a constant population size coalescent tree prior were used. The MCMC was run at 50,000,000 generations and sampled at every 5000 generations. The effective sample size (ESSs) of the analysis was checked by using the software Tracer v1.6 (http://tree.bio.ed.ac.uk/software/tracer/, accessed on 12 June 2022). The MCC host discrete traits output tree was generated by using TreeAnnotator v1.10.4 (http://tree.bio.ed.ac.uk/software/beast/, accessed on 12 June 2022) afterburn 10% of the first trees. The host phylogenetic tree was reconstructed by using the software FigTree v.1.4.3 (http://tree.bio.ed.ac.uk/, accessed on 12 June 2022) [21]. 

### 2.5. Anti-HEV Antibody Detection from Sheep and Pigs

Serum samples from sheep (*n* = 42) and pigs (*n* = 45) collected in the study area were investigated for antibodies to HEV. In-house ELISA was used in this study as previously described [21]. Anti-HEV antibodies were detected by enzyme-linked immunosorbent assay (ELISA) using VLPs derived from HEV genotype 3 as antigens. For the detection of antigen-bound IgG, an anti-pig IgG antibody-HRP conjugate and an anti-sheep IgG antibody-HRP conjugate (Bethyl Laboratories Inc., Montgomery, TX, USA) were applied as a secondary antibody according to the method described in a previous report [21]. The serum samples were diluted 1:100 with PBS containing 0.05% Tween 20, and 10% of Block Ace (DS Pharma Promo Co., Ltd., Osaka, Japan) and incubated for 1 h at room temperature. After the secondary antibody reactions, 50 μL of TMB (3,3′,5,5′-tetramethylbenzidine) (Kirkegaard & Perry Laboratories Inc., Baltimore, MD, USA) was added, and after 10-min incubation at room temperature, 50 μL of 2 M sulfuric acid was added to stop the reactions. The optical density (OD) value at 450 nm was measured by a microplate spectrophotometer (Thermo Fisher Scientific Inc., Waltham, MA, USA). Positive control sera were obtained from pigs experimentally infected with HEV, and negative sera were obtained from non-infected, healthy pigs. The cut-off value was set at 0.295 by calculating the average value + 2 standard deviation of the negative samples (*n* = 5). The sensitivity and specificity of this ELISA test in 20 experimentally infected and 24 negative pigs were 90.0% (95% CI: 68.30–98.77) and 91.67% (95% CI: 73.0–98.97), respectively.

### 2.6. Statistical Analysis

The age of the pigs was categorized as piglet less than 6 months; older than 6 months; over 1 year old. All epidemiological data were triple-entered and cleaned in Microsoft Office Excel 2010 (Microsoft Co., Redmond, WA, USA). We performed univariable and multivariable analysis using IBM SPSS Statistics 26 (IBM, Chicago, IL, USA) and Fisher’s exact test calculation [25]. Categorical variables were compared using Fisher’s exact test and χ^2^ test. A statistically significant difference was considered when the *p*-values were <0.05. The adjusted odds ratio (OR) and the 95% confidence interval (CI) for HEV risk factors were analyzed using univariate and multivariable logistic regression. 

## 3. Results

### 3.1. Prevalence of HEV RNA in Sheep in Töv Province, Mongolia

All of the fecal and liver samples were examined for the HEV ORF2 region using nested RT-PCR. The HEV ORF2 region was detected in sheep liver and fecal samples (Table 1). A total of 3/60 (5%) positive sheep livers were found near farms, which is in Bayanchandmani soum, Töv Province. Three RT-PCR-positive sheep liver samples were sequenced, and these sequences belonged to genotype 4 (accession numbers LC752752, LC752753, and LC702420). The sequence was highly homologous (98%) to those detected from pig samples (PF121, PF101, and PF175, which is located in the Songino Khairkhan district of Ulaanbaatar city; accession numbers LC413554, LC413553, and LC413557) (Table 1). The HEV prevalence in the feces samples was 2% (4/200) in sheep. 

### 3.2. Prevalence of HEV RNA among Pigs in Töv Province, Mongolia

Pig feces (PF) and pig livers (PL) were collected from a farm (sample numbers: MGL-PF 1-200, MGL-PL 1-60). A total of 200 pig feces samples and 60 pig liver samples were examined for the HEV ORF2 region using nested RT-PCR, and the HEV ORF2 regions were detected in fecal samples (30/200, 15%) and liver samples (4/60, 6.6%) using nested RT-PCR (Table 2). 

### 3.3. Genetic Analysis of the HEV from Pigs and Sheep 

**Figure 2:**
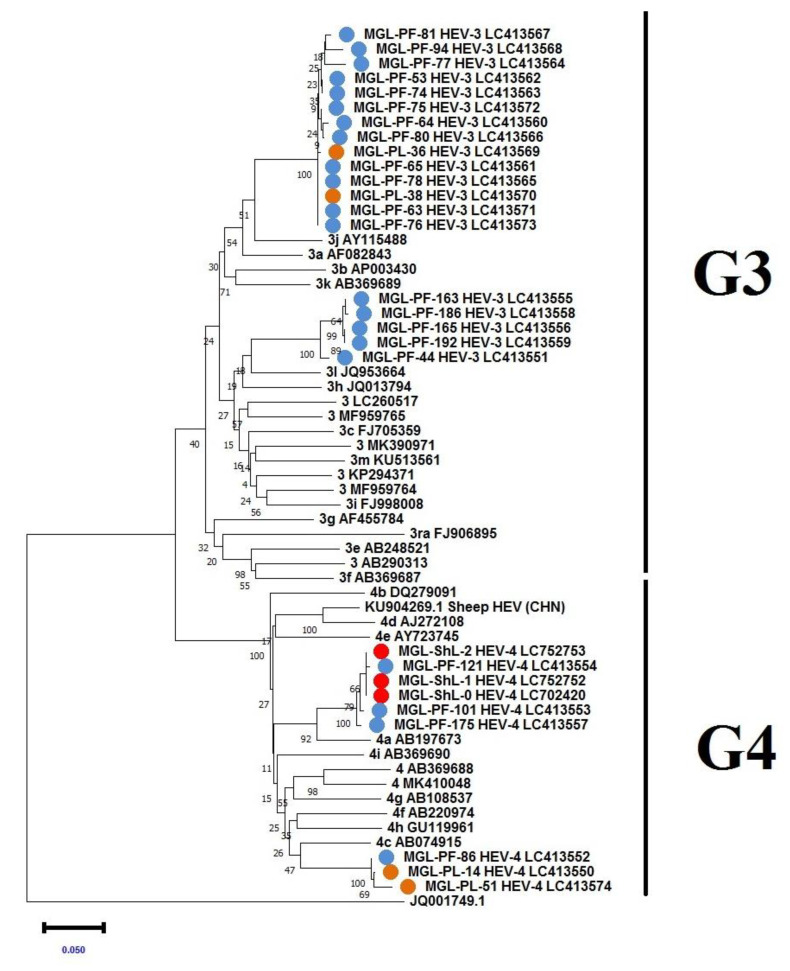
Phylogenetic analysis of HEV-ORF2 regions detected in animals in Mongolia with Smith’s prototype sequences. Phylogenetic tree (neighbor-joining method) of the open reading frame (ORF) 2 gene region of hepatitis E virus (HEV) genotypes 3 and 4. The numbers and letters before the accession number in the table indicate the HEV subtype, and the genotype (HEV-3, HEV-4) is shown after the sample number identified in this study. Blue dots represent pig feces samples, orange dots represent pig liver samples, and red dots represent sheep liver samples (ShL-0-2). A total of 28 samples were sequenced for HEV ORF2. MEGA X was used for multiple alignments and phylogenetic tree construction. Based on the phylogenetic tree results, 9/28 sequences belonged to HEV-4, whereas 19/28 sequences belonged to HEV-3. The ORF2 gene region of the JQ001749.1_Bat HEV/GE/2009 (GE—Germany) complete genome was used as an outgroup. The bar represents 0.05 nucleotide substitutions per site.

**Figure 3:**
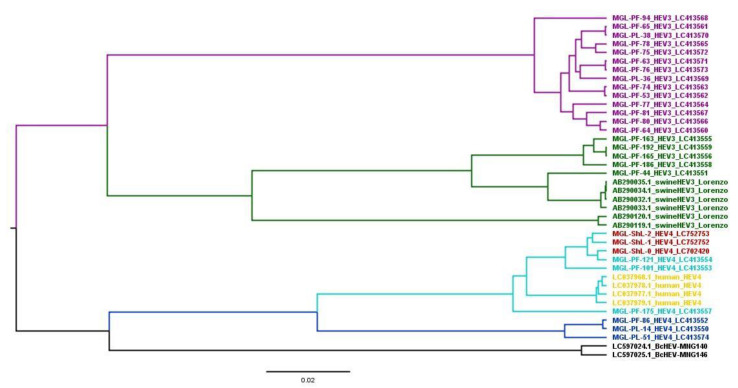
The Bayesian maximum clade credibility (MCC) host discrete traits tree for 302 bp HEV ORF2 nucleotide sequences (nt position 6022–6324) of 40 Mongolian strains, including 28 sequences from our study and reference strains (six swine HEV-ORF2 genotype 3, reprinted with permission from reference [16], four human HEV-ORF2 sequences [19], and two Bactrian camels HEV-ORF2 (BcHEV1, BcHEV2) sequences [26]) obtained from the GenBank database. The phylogenetic host tree indicated transmission between HEV hosts, the host at the node indicated the ancestor of the sub-group, and the number at the node indicated the posterior probability. The bar at the bottom of the figure denotes evolutionary distance.

The nucleotide sequence analysis of the HEV ORF2 region detected in pigs identified genotypes 3 and 4. The pig-derived HEVs belonging to genotype 3 were divided into two clusters. The viruses belonging to genotype 4 were divided into two clusters, one of which showed high homology to the sheep sequence (Supplementary Tables). The accession numbers of the HEV-3 type were LC413551, LC413555, LC413556, LC413558, and LC413573. The accession numbers of the HEV-4 type were LC413550, LC413552, LC413553, LC413554, LC413557, and LC413574. The HEV detection rate for those older than 6 months was 12.3%, with 75% of the positive samples belonging to genotype 3 and 25% belonging to genotype 4. Pigs over 1 year of age were slaughtered and RT-PCR for HEV in the liver was performed. The results showed that 6.6% (4/60) of the samples were positive and that the positive samples belonged to genotypes 3 and 4, with two animals in each (accession numbers LC413570, LC413569, LC413550, and LC413574) (Table 2, Supplementary Tables). Phylogenetic analysis showed that 19 out of the 25 sequences belonged to genotype 3 (Figure 2 and Figure 3).

The sequence analysis of the ORF2 region of three of the HEV-detected sheep belonged to genotype 4 (accession numbers: LC702420, LC752752, and LC752753). The sequence was identical to the genotypes (LC413553, LC413554, and LC413557) detected in pigs from the same region. Their amino sequences were also 99% identical to a previously reported sequence (ABO93596). Homology comparison of the sequences of HEV genotype 4 identified in Mongolian sheep and China (KU904269) showed a homology of 86%. Therefore, the HEV genotype 4 identified in Mongolian sheep was different from the HEV genotype 4 in China.

The Bayesian MCC hosts a discrete traits tree for 302 bp HEV ORF2 nucleotide sequences (nt position 6022–6324) of a total of 40 Mongolian strains, including 28 sequences from our study and reference strains (six swine HEV-ORF2 sequences, genotype 3 [16], four human HEV-ORF2 sequences [19], and two Bactrian camel HEV-ORF2 (BcHEV1, BcHEV2) sequences [26]) obtained from the GenBank database (Figure 3). The phylogenetic host tree indicated transmission between HEV hosts, the host at the node indicated the ancestor of the sub-group, and the number at the node indicated the posterior probability. Genetic analysis of HEV determined in Mongolian people suggested that it was derived from genotype 4 of HEV in pigs and sheep in that country. In addition, six swine HEV-ORF2 sequences, three sheep HEV-ORF2 from our study, and four human HEV-ORF2 sequences from a previous study [19] belong to genotype 4 of HEV. The other 25 pig HEV-ORF2 sequences belong to genotype 3, including 19 sequences from our study and 6 sequences from F. Lorenzo’s study [16].

### 3.4. Anti-HEV Antibody Detection from Sheep and Pigs

Serum samples from sheep (*n* = 42) and pigs (*n* = 45) collected in the study area were investigated for anti-HEV IgG. HEV-3 genotype VLP protein was used as the ELISA antigen. The HEV seropositive animals were 11.9% (5/42) of the sheep and 35.5% (16/45) of the pigs. 

### 3.5. Statistical Results

Univariable analysis was performed (Supplementary Table S3). Categorical variables were compared using Fisher’s exact test and χ^2^ test. Age and foreign introduction of pigs are statistically significant (*p* < 0.05), while gender and feeding source is not statistically significant. A risk assessment was performed for each variable (Table 3). The odds ratio between the age ranges was calculated when the age of 1 year old is the reference, and the highest was a less than 6 months old piglet, OR = 3.42. The odds ratio between the feeding source was calculated when mill offal is the reference; the highest OR equals 2.6 in the small ruminant offal. The odds ratio between whether a foreign introduction is introduced or not is the reference, the highest OR equals 3.06 in the foreign introduction. These factors led to the higher risk for HEV cases in Mongolia. These three risk factors were statistically significant (*p* < 0.02–0.003, 95% CI). Gender was not statistically significant.

## 4. Discussion

HEV has been detected in pigs and other animals all over the world including in Asia [6]. The HEV genotypes predominantly detected in Asia are genotypes 3 and 4, which are known to cause zoonoses. In this study, we used molecular methods to demonstrate the prevalence of HEV in Mongolian pigs and sheep. HEV genotype 3 has been detected in Mongolia previously [16]; however, the sequences of the viruses obtained in this study were different from those previously reported. Moreover, our results have shown the efficient detection of HEV RNA in feces. Virus shedding is an important factor in virus transmission among pigs on farms [27]. Thus, these results imply that sanitary control on pig farms is an important issue for healthy livestock management in the country. Since meat is the staple diet of the Mongolian people, there is concern that meat contaminated with HEV could cause adverse situations, especially for pregnant women and other hepatitis virus carriers. The prevalence of HEV in the swine liver requires caution for handlers at slaughterhouses and in meat processing factories. The phylogenetic tree analysis showed that genotypes 3 and 4 were detected in pigs. The HEV gene sequence detected in pigs in Mongolia is highly homologous to previously reported cases, which may be due to the establishment of the virus based on the importation of live pigs to Mongolia. The history of live piglet imports from China suggests that Mongolian HEVs originate from two different sources. HEV genotypes 3 and 4 are similar to Asian isolates, including those from Japan and China [28]. One HEV-4 genotype cluster was also identified in sheep-derived HEVs, suggesting that HEVs can be transmitted between pigs and sheep. 

From the results of the phylogenetic analysis, 19 sequences belonged to cluster HEV-3 and six sequences belonged to cluster HEV-4. The HEV strain may be transmitted between farms via human and pig movement. Pigs are usually fed on the by-products of slaughterhouse-derived sheep’s internal organs. The pigs had no restrictions on their movement. Phylogenetic analysis of the ORF2 region revealed that the HEV genotypes in the infected pigs in the vicinity of Ulaanbaatar city are homologous to those in sheep. This could be because sheep co-graze near pig farms in the same area. In comparison with HEVs from sheep detected in Töv Province, phylogenetic analysis showed genetic similarity with PF121, PF101, and PF175 from pigs. The geographical distance between Bayanchandmani soum (the sheep sample area) and Songino Khairkhan district intersects with livestock movement and feed distribution. Therefore, it is quite possible that HEV of livestock origin between the same regions could be transmitted via various routes. Mongolian sheep and goats usually co-graze in pasturelands together with other livestock, including cattle. We assume that sheep are not HEV reservoirs but are spillover infected animals. Sheep are most likely spillover infected animals due to HEV infection from pigs. The maximum clade credibility tree analysis in this study suggests that sheep HEV is of porcine origin. Chinese and Italian researchers have detected HEV in fecal and milk samples from goats and sheep [2,9,29]. HEV isolates have been detected in the southern and eastern parts of China, including Yunnan Province, Tai’an region, and Shandong Province [2,10,30]. 

With regard to HEV detection efficiency and the age of the tested pigs, the detection rate was low in subjects aged approximately 1 year old but high in younger populations (less than 6 months old) [27]. Fecal shedding of the virus is frequently detected in young pigs under 6 months of age and this age is a risk factor for environmental shedding of the virus. On the other hand, since the virus is detected in the liver even at the age of over 6 months, there is a risk of HEV infection in the livers of pigs shipped to the market. The main concern is that the virus shed from infected pigs is transmitted to other pigs. Each year, 10–20% of pigs are replaced by foreign introductions, mainly from China. Fattened pigs are slaughtered for meat, and piglets are fattened on farms. The statistical analysis indicated that the foreign introduction of pigs was a strong factor and that another possible cause of infection could be the feeding source. Except for piglets, all of the pigs were fed with mill offal as a fattening food. According to a cross-sectional study, there was a statistically significant difference in terms of the use of small ruminant offal and mill offal. Free-range pigs can contaminate the water or hay resources of sheep that graze near pig farms. Gender was not found to be associated with the risk estimation analysis. 

Improvements are needed to reduce the opportunities for contact with other livestock in the future. In our study, HEV-antibody-positive sheep were detected (positivity at about 11.9% (5/42) in sheep and 35.5% (16/45) in pigs), and previous reports of HEV infection epidemiology around the world also suggest that ruminant livestock is at risk of HEV infection [7,31,32,33,34,35,36]. If there is the contamination of HEV from the internal organs of livestock, the possible transmission risk of the virus in Mongolia will be high. Mongolian people’s main source of food is meat. HEV-contaminated food can lead to bad situations, especially in pregnant women and other hepatitis virus carriers. HEV has been reported to have been detected in sheep from neighboring China. Anti-HEV antibodies were detected in 35.2% of sera and 57.7% of slaughter meat samples from Xinjiang, which borders the Mongolian west border. All of the isolates belonged to genotype 4 [5]. Moreover, various types of livestock are mixed-reared in China, and it has been pointed out that HEVs are transmitted between livestock [10]. To date, sheep infected with HEV-4 have been reported from China as follows: KU904269 [30] sheep HEV. In our study, the MCC analysis suggested that the pig and sheep HEV detected in this study could be transmitted to humans, presenting a public health issue. A detailed investigation is required for virus transmission among livestock, and investigation and management of HEV-infected animals are also important for public health. The risk verification of the use of pig feed from Mongolian livestock would be a further research point from the perspective of zoonotic diseases. 

## 5. Conclusions

The present results suggest that the spread of HEV in Mongolian livestock is a transmission risk owing to the management of introductions among pigs. Furthermore, HEV of the same genotype was shared among pigs and sheep reared in the same area. As sheep offal is fed to pigs, livestock husbandry management to interrupt HEV transmission and prevent its spread appears necessary. The virus was also confirmed in the livers of slaughtered pigs and sheep, suggesting the need for a call to attention with regard to food hygiene. These data show that measures are needed to improve prevention and control strategies for food safety.

## Figures and Tables

**Table 1:** Detection of HEV RNA of sheep in Töv Province.

Samples	Numbers	Positive Nos.	Rate (%)	95% CI
Liver	60	3	5.0	4.75–5.25%
Feces	200	4	2.0	1.25–2.75%

**Table 2:** Detection of HEV RNA on pig farms.

Pig Age	Male/Female	Positive in Feces	Positive in the Liver	95% CI
Less than 6 months	10/30	10/40 (25%)	NT	20–30%
Older than 6 months	32/98	16/130 (12,3%)	NT	7.3–17.3%
Around 1 year	8/22	4/30 (13.3%)	NT	8.3–18.3%
Over 1 year	15/45	NT	4/60 (6.6%)	1.6–11.6%
Total	65/195	30/200 (15%)	4/60 (6.6%)	

NT: not tested.

**Table 3:** Risk factor analysis of HEV in farm pigs in Ulaanbaatar city.

Variables	Category	Total	Positive	Negative	Positive (%)	OR	*p*-Value
Sex	Female	195	31	164	15.9	1.86	0.183
	Male	65	6	59	9.2	Ref	
Age	Less than 6 months	40	10	30	25	3.42	0.014
	More than 6 months	130	16	114	12.3	1.44	0.424
	1 year old	90	8	82	8.9	Ref	
Feeding source	Sheep offal	40	10	30	25	2.6	0.02
	Mill offal	220	25	195	11.4	Ref	
Foreign introduction	Yes	51	13	38	25.5	3.06	0.003
	No	209	21	188	10	Ref	

## Data Availability

The datasets presented in this study are publicly available.

## Supplementary Materials

The following supporting information can be downloaded at: https://www.mdpi.com/article/10.3390/ani13050891/s1, Table S1: Sample genotype and HEV homology % (NA) detected in this study; Table S2: Sample genotype and HEV homology % (AA) detected in this study; Table S3: Univariate analysis of HEV in farm pig in Ulaanbaatar city; Table S4: HEV genotypes used in this phylogenetic tree; Table S5: Reference Information of Research Pigs; Table S6: Reference Information of Research Sheep.
