# Supplementary material for: Hepatitis E Virus (HEV) Spreads from Pigs and Sheep in Mongolia
Source: Animals (Basel). 2023 Mar 1;13(5):891. doi: 10.3390/ani13050891 (PMC10000034; doi:10.3390/ani13050891)
Supplement: Supplementary file 1 [file animals-13-00891-s001.zip › animals-2249112-supplementary.pdf]

Supplementary Material

Supplementary Table S1.
Sample genotype and HEV homology % (NA) detected in this study

No	Sample name	Genotype	Accession number	NA homology %	Compared sequence Acc. No.
1	MGL-PL-14	4	LC413550	297/302(98%)	AB481227.1
2	MGL-PF-44	3	LC413551	296/301(98%)	AB290312.1
3	MGL-PF-86	4	LC413552	298/302(99%)	AB481227.1
4	MGL-PF-101	4	LC413553	295/302(98%)	LC037968.1
5	MGL-PF-121	4	LC413554	294/302(97%)	LC037968.1
6	MGL-PF-163	3	LC413555	296/301(98%)	AB290106.1
7	MGL-PF-165	3	LC413556	296/301(98%)	AB290106.1
8	MGL-PF-175	4	LC413557	296/302(98%)	LC037968.1
9	MGL-PF-186	3	LC413558	295/301(98%)	AB290106.1
10	MGL-PF-192	3	LC413559	296/301(98%)	AB290106.1
11	MGL-PF-64	3	LC413560	296/301(98%)	AB481228.1
12	MGL-PF-65	3	LC413561	299/301(99%)	AB471965.1
13	MGL-PF-73	3	LC413562	298/301(99%)	AB481228.1
14	MGL-PF-74	3	LC413563	298/301(99%)	AB481228.1
15	MGL-PF-77	3	LC413564	293/301(97%)	AB471965.1
16	MGL-PF-78	3	LC413565	297/301(99%)	AB470987.1
17	MGL-PF-80	3	LC413566	297/301(99%)	AB471965.1
18	MGL-PF-81	3	LC413567	295/301(98%)	AB481228.1
19	MGL-PF-94	3	LC413568	294/301(98%)	AB481228.1
20	MGL-PL-36	3	LC413569	298/301(99%)	AB481228.1
21	MGL-PL-38	3	LC413570	299/301(99%)	AB481228.1
22	MGL-PF-63	3	LC413571	299/301(99%)	AB471965.1
23	MGL-PF-75	3	LC413572	298/301(99%)	AB471965.1
24	MGL-PF-76	3	LC413573	299/301(99%)	AB481228.1
25	MGL-PL-51	4	LC413574	295/302(98%)	AB481227.1
26	MGL-ShL-0	4	LC702420	295/302(98%)	LC037968.1
27	MGL-ShL-1	4	LC752752	295/302(98%)	LC037968.1
28	MGL-ShL-2	4	LC752753	295/302(98%)	LC037968.1

Supplementary Table S2.
Sample genotype and HEV homology % (AA) detected in this study

No	Sample name	Genotype	Accession number	AA homology %	Compared sequence Acc. No.
1	MGL-PL-14	4	LC413550	204/204 (99%)	AHK60815.1
2	MGL-PF-44	3	LC413551	202/202 (100%)	QLE10601.1
3	MGL-PF-86	4	LC413552	206/206 (100%)	AHK60817.1
4	MGL-PF-101	4	LC413553	205/205 (100%)	QIL87290.1
5	MGL-PF-121	4	LC413554	200/200 (99%)	CAC51491.1
6	MGL-PF-163	3	LC413555	203/203 (100%)	BAF65183.1
7	MGL-PF-165	3	LC413556	203/203 (100%)	BAF65117.1
8	MGL-PF-175	4	LC413557	205/205 (100%)	QIL87290.1
9	MGL-PF-186	3	LC413558	203/203 (100%)	BAF65183.1
10	MGL-PF-192	3	LC413559	203/203 (100%)	BAF65183.1
11	MGL-PF-64	3	LC413560	202/202 (99%)	AFY26248.1
12	MGL-PF-65	3	LC413561	202/202 (100%)	QLE10601.1
13	MGL-PF-73	3	LC413562	202/202 (100%)	QLE10601.1
14	MGL-PF-74	3	LC413563	202/202 (100%)	QLE10601.1
15	MGL-PF-77	3	LC413564	196/196 (98%)	AKH15687.1
16	MGL-PF-78	3	LC413565	202/202 (100%)	QLE10601.1
17	MGL-PF-80	3	LC413566	202/202 (99%)	AFY26248.1
18	MGL-PF-81	3	LC413567	201/201 (98%)	QBO24257.1
19	MGL-PF-94	3	LC413568	198/198 (98%)	ALH23992.1
20	MGL-PL-36	3	LC413569	202/202 (100%)	QLE10601.1
21	MGL-PL-38	3	LC413570	202/202 (100%)	QLE10601.1
22	MGL-PF-63	3	LC413571	202/202 (100%)	QLE10601.1
23	MGL-PF-75	3	LC413572	202/202 (99%)	QLE10601.1
24	MGL-PF-76	3	LC413573	202/202 (100%)	QLE10601.1
25	MGL-PL-51	4	LC413574	199/199 (97%)	AUH25459.1
26	MGL-ShL-0	4	LC702420	198/198 (99%)	ABO93596
27	MGL-ShL-1	4	LC752752	198/198 (99%)	ABO93596
28	MGL-ShL-2	4	LC752753	198/198 (99%)	ABO93596

Supplementary Table S3.
Univariate analysis of HEV in farm pig in Ulaanbaatar city.

Variable	Animals	Total	Positive	Negative	Positive (%)	Fisher's Exact Test (2x3) p-value	Chi-square test (2x2) p-value	Statistical significance 95% CI
Sex	Female	195	31	164	15.9	-	0.832	Not significant
	Male	65	6	59	9.2			
Age	Less than 6 months	40	10	30	25	0.048	-	Significant
	Elder than 6 months	130	16	114	12.3			
	1 year old and adults	90	8	82	8.9			
Feeding source	Sheep offal	40	10	30	25	-	0.055	Not significant
	Mill offal	220	25	195	11.4			
Foreign introduction	yes	51	13	38	25.5	-	0.0001	Significant
	no	209	21	188	10			

Supplementary Table S4.
HEV genotypes used in this phylogenetic tree

No	References (*)	Accession number
1	Hepatitis E virus gene for capsid protein, partial cds, isolate: HE-JA16-0509_ORF2-cln16	LC202065.1(JPN)
2	Swine hepatitis E virus ORF2 gene for capsid protein, partial cds, isolate: swJ17-1	AB094272.1(JPN)
3	Hepatitis E virus genomic RNA, nearly complete genome, isolate: JDEER-Hyo03L, ORF2	AB189071.1(JPN)
4	Swine hepatitis E virus for capsid protein, partial cds, isolate: swJTY1-1, ORF2	AB194523.1(JPN)
5	Hepatitis E virus isolate EChN22 capsid protein gene, partial cds, ORF2	HM439285(CHN)
6	Hepatitis E virus ORF2 gene, partial cds, clone: wbJSG1-L2	AB180053.1(JPN)
7	Hepatitis E virus strain L1HT capsid gene, partial cds, ORF2	KC145147.1(SPN)
8	Hepatitis E virus isolate UIAS268 capsid protein (ORF2) gene, partial cds	DQ061078(RUS)
9	Hepatitis E virus isolate Rec2S6A SG capsid protein (ORF2) gene, partial cds, ORF2	MK140443.1(UK)
10	Swine hepatitis E virus isolate SA28 capsid protein gene, partial cds, ORF2	FJ998016(GER)
11	Swine hepatitis E virus isolate SA13 capsid protein gene, partial cds, ORF2	FJ998014(GER)
12	Swine hepatitis E virus isolate SA11 capsid protein gene, partial cds, ORF2	FJ998013(GER)
13	Hepatitis E virus gene for capsid protein, partial cds, isolate: rbIM213L, ORF2	AB741261 (CHN-IM)
14	Hepatitis E virus gene for capsid protein, partial cds, isolate: rbIM226S, ORF2	AB741258 (CHN-IM)
15	Hepatitis E virus isolate GDC9, complete genome, ORF2	FJ906895.1(CHN)
16	Hepatitis E virus isolate Ch-S-12 nonfunctional capsid protein gene, partial sequence, ORF2	EF100130(CHN)
17	Hepatitis E virus gene for capsid protein, partial cds, isolate: wbJGF_06-1-L2, ORF2	AB605222(JPN)
18	Orthohepevirus A gene for capsid protein, partial cds, isolate: rbIM205, ORF2	AB741249 (CHN-IM)
19	Hepatitis E virus gene for capsid protein, partial cds, isolate: HE-JTB98-2, ORF2	AB292654(JPN)
20	Orthohepevirus A gene for capsid protein, partial cds, isolate:rbIM198, ORF2	AB741245 (CHN-IM)

21	Orthohepevirus A gene for capsid protein, partial cds, isolate: rbIM137-c2, ORF2	AB741195 (CHN-IM)
22	Hepatitis E virus isolate Ch-S-8 capsid protein gene, partial cds, ORF2	EF100126(CHN)
23	Orthohepevirus A genomic RNA, complete genome, isolate: JBOAR135-Shiz09, ORF2	AB573435.2(JPN)
24	Hepatitis E virus isolate SD-YANTAIhev03 capsid protein gene, partial cds, ORF2	KU904269.1(CHN)
25	Hepatitis E virus (genotype 4) complete genome sequence, ORF2	AJ272108.1(CHN)
26	Hepatitis E virus ORF2 gene for capsid protein, partial cds, isolate: HRC-HE1, genotype: 4	AB434132(JPN)
27	Hepatitis E virus ORF2 gene for capsid protein, partial cds, isolate: HRC-HE6, genotype: 4	AB434137(JPN)
28	Hepatitis E virus gene for capsid protein, partial cds, isolate: S6_wt, ORF2	AB525045(JPN)
29	Hepatitis E virus isolate SDTAhev01 capsid protein mRNA, partial cds, ORF2	MF443448.1(CHN)
30	Hepatitis E virus isolate SDTAhev02 capsid protein mRNA, partial cds, ORF2	MF443449.1(CHN)
31	Human hepatitis E virus genotype 2a strain Mexican nonstructural polyprotein, structural viral protein, and structural polyprotein genes, complete cds, ORF2	M74506.1 (MEX)
32	Hepatitis E virus, complete genome, ORF2	NC_001434.1(CHN)
33	Hepatitis E virus isolate MO, complete genome, ORF2	JQ655733(CHN)
34	Orthohepevirus D isolate BatHEV/BS7/GE/2009, ORF2, outgroup	JQ001749.1(GER)
35	MGL-PL-14 Mongolian isolate pig liver, ORF2	LC413550 (MGL)
36	MGL-PF-44 Mongolian isolate pig faeces, ORF2	LC413551 (MGL)
37	MGL-PF-86 Mongolian isolate pig faeces, ORF2	LC413552 (MGL)
38	MGL-PF-101 Mongolian isolate pig faeces, ORF2	LC413553 (MGL)
39	MGL-PF-121 Mongolian isolate pig faeces, ORF2	LC413554 (MGL)
40	MGL-PF-163 Mongolian isolate pig faeces, ORF2	LC413555 (MGL)
41	MGL-PF-165 Mongolian isolate pig faeces, ORF2	LC413556 (MGL)
42	MGL-PF-175 Mongolian isolate pig faeces, ORF2	LC413557 (MGL)
43	MGL-PF-186 Mongolian isolate pig faeces, ORF2	LC413558 (MGL)
44	MGL-PF-192 Mongolian isolate pig faeces, ORF2	LC413559 (MGL)
45	MGL-PF-64 Mongolian isolate pig faeces, ORF2	LC413560 (MGL)
46	MGL-PF-65 Mongolian isolate pig faeces, ORF2	LC413561 (MGL)
47	MGL-PF-73 Mongolian isolate pig faeces, ORF2	LC413562 (MGL)

48	MGL-PF-74 Mongolian isolate pig faeces, ORF2	LC413563 (MGL)
49	MGL-PF-77 Mongolian isolate pig faeces, ORF2	LC413564 (MGL)
50	MGL-PF-78 Mongolian isolate pig faeces, ORF2	LC413565 (MGL)
51	MGL-PF-80 Mongolian isolate pig faeces, ORF2	LC413566 (MGL)
52	MGL-PF-81 Mongolian isolate pig faeces, ORF2	LC413567 (MGL)
53	MGL-PF-94 Mongolian isolate pig faeces, ORF2	LC413568 (MGL)
54	MGL-PL-36 Mongolian isolate pig liver, ORF2	LC413569 (MGL)
55	MGL-PL-38 Mongolian isolate pig liver, ORF2	LC413570 (MGL)
56	MGL-PF-63 Mongolian isolate pig faeces, ORF2	LC413571 (MGL)
57	MGL-PF-75 Mongolian isolate pig faeces, ORF2	LC413572 (MGL)
58	MGL-PF-76 Mongolian isolate pig faeces, ORF2	LC413573 (MGL)
59	MGL-PL-51 Mongolian isolate pig liver, ORF2	LC413574 (MGL)
60	MGL-ShL-0 Mongolian isolate sheep liver, ORF2	LC702420 (MGL)
61	MGL-ShL-1 Mongolian isolate sheep liver, ORF2	LC752752 (MGL)
62	MGL-ShL-2 Mongolian isolate sheep liver, ORF2	LC752753 (MGL)

Supplementary Table S5.
Reference Information of Research Pigs

№	Sex	Age	feeding_ source	HEV	foreign introduction	sample
1	1	2	1	1	0	feces
2	2	2	1	1	0	feces
3	2	2	1	1	0	feces
4	2	2	1	1	0	feces
5	1	2	1	0	0	feces
6	2	2	1	0	0	feces
7	2	2	1	0	0	feces
8	2	2	1	0	0	feces
9	1	2	1	0	0	feces
10	2	2	1	0	0	feces
11	2	2	1	0	0	feces
12	2	2	1	0	0	feces
13	1	2	1	0	0	feces
14	2	2	1	0	0	feces
15	2	2	1	0	0	feces
16	2	2	1	0	0	feces
17	1	2	1	0	0	feces
18	2	2	1	0	0	feces
19	2	2	1	0	0	feces
20	2	2	1	0	0	feces
21	1	2	1	0	0	feces
22	2	2	1	0	0	feces
23	2	2	1	0	0	feces
24	2	2	1	0	0	feces
25	1	2	1	0	0	feces
26	2	2	1	0	0	feces
27	2	2	1	0	0	feces
28	2	2	1	0	0	feces
29	1	2	1	0	0	feces
30	2	2	1	0	0	feces
31	2	0	0	1	1	feces
32	2	0	0	1	1	feces
33	1	0	0	1	1	feces
34	2	0	0	1	1	feces
35	2	0	0	1	1	feces
36	2	0	0	1	1	feces

37	1	0	0	1	0	feces
38	2	0	0	1	0	feces
39	2	0	0	1	0	feces
40	2	0	0	0	1	feces
41	1	0	0	0	1	feces
42	2	0	0	0	1	feces
43	2	0	0	0	1	feces
44	2	0	0	0	1	feces
45	1	0	0	0	1	feces
46	2	0	0	0	1	feces
47	2	0	0	0	1	feces
48	2	0	0	0	1	feces
49	1	0	0	0	1	feces
50	2	0	0	0	1	feces
51	2	0	0	0	1	feces
52	2	0	0	0	1	feces
53	1	0	0	0	0	feces
54	2	0	0	0	0	feces
55	2	0	0	0	0	feces
56	2	0	0	0	0	feces
57	1	0	0	0	0	feces
58	2	0	0	0	0	feces
59	2	0	0	0	0	feces
60	2	0	0	0	0	feces
61	1	0	0	0	0	feces
62	2	0	0	0	0	feces
63	2	0	0	0	0	feces
64	2	0	0	0	0	feces
65	1	0	0	0	0	feces
66	2	0	0	0	0	feces
67	2	0	0	0	0	feces
68	2	0	0	0	0	feces
69	1	0	0	0	0	feces
70	2	0	0	0	0	feces
71	2	1	1	1	1	feces
72	2	1	1	1	1	feces
73	1	1	1	1	1	feces
74	2	1	1	1	1	feces
75	2	1	1	1	1	feces

76	2	1	1	1	1	feces
77	1	1	1	1	1	feces
78	2	1	1	1	1	feces
79	2	1	1	1	1	feces
80	2	1	1	1	1	feces
81	1	1	1	1	1	feces
82	2	1	1	1	0	feces
83	2	1	1	1	0	feces
84	2	1	1	1	0	feces
85	1	1	1	1	0	feces
86	2	1	1	1	0	feces
87	2	1	1	1	0	feces
88	2	1	1	0	0	feces
89	1	1	1	0	0	feces
90	2	1	1	0	0	feces
91	2	1	1	0	0	feces
92	2	1	1	0	0	feces
93	1	1	1	0	0	feces
94	2	1	1	0	0	feces
95	2	1	1	0	0	feces
96	2	1	1	0	0	feces
97	1	1	1	0	0	feces
98	2	1	1	0	0	feces
99	2	1	1	0	0	feces
100	2	1	1	0	0	feces
101	1	1	1	0	0	feces
102	2	1	1	0	0	feces
103	2	1	1	0	0	feces
104	2	1	1	0	0	feces
105	1	1	1	0	0	feces
106	2	1	1	0	0	feces
107	2	1	1	0	0	feces
108	2	1	1	0	0	feces
109	1	1	1	0	0	feces
110	2	1	1	0	0	feces
111	2	1	1	0	0	feces
112	2	1	1	0	0	feces
113	1	1	1	0	0	feces
114	2	1	1	0	0	feces
115	2	1	1	0	0	feces

116	2	1	1	0	0	feces
117	1	1	1	0	0	feces
118	2	1	1	0	0	feces
119	2	1	1	0	0	feces
120	2	1	1	0	0	feces
121	1	1	1	0	0	feces
122	2	1	1	0	0	feces
123	2	1	1	0	0	feces
124	2	1	1	0	0	feces
125	1	1	1	0	0	feces
126	2	1	1	0	0	feces
127	2	1	1	0	0	feces
128	2	1	1	0	0	feces
129	1	1	1	0	0	feces
130	2	1	1	0	0	feces
131	2	1	1	0	0	feces
132	2	1	1	0	0	feces
133	1	1	1	0	0	feces
134	2	1	1	0	0	feces
135	2	1	1	0	0	feces
136	2	1	1	0	0	feces
137	1	1	1	0	0	feces
138	2	1	1	0	0	feces
139	2	1	1	0	0	feces
140	2	1	1	0	0	feces
141	1	1	1	0	0	feces
142	2	1	1	0	0	feces
143	2	1	1	0	0	feces
144	2	1	1	0	0	feces
145	1	1	1	0	0	feces
146	2	1	1	0	0	feces
147	2	1	1	0	0	feces
148	2	1	1	0	0	feces
149	1	1	1	0	0	feces
150	2	1	1	0	0	feces
151	2	1	1	0	0	feces
152	2	1	1	0	0	feces
153	1	1	1	0	0	feces
154	2	1	1	0	0	feces

155	2	1	1	0	0	feces
156	2	1	1	0	0	feces
157	1	1	1	0	0	feces
158	2	1	1	0	0	feces
159	2	1	1	0	0	feces
160	2	1	1	0	0	feces
161	1	1	1	0	0	feces
162	2	1	1	0	0	feces
163	2	1	1	0	0	feces
164	2	1	1	0	0	feces
165	1	1	1	0	0	feces
166	2	1	1	0	0	feces
167	2	1	1	0	0	feces
168	2	1	1	0	0	feces
169	1	1	1	0	0	feces
170	2	1	1	0	0	feces
171	2	1	1	0	0	feces
172	2	1	1	0	0	feces
173	1	1	1	0	0	feces
174	2	1	1	0	0	feces
175	2	1	1	0	0	feces
176	2	1	1	0	0	feces
177	1	1	1	0	0	feces
178	2	1	1	0	0	feces
179	2	1	1	0	0	feces
180	2	1	1	0	0	feces
181	1	1	1	0	0	feces
182	2	1	1	0	0	feces
183	2	1	1	0	0	feces
184	2	1	1	0	0	feces
185	1	1	1	0	0	feces
186	2	1	1	0	0	feces
187	2	1	1	0	0	feces
188	2	1	1	0	0	feces
189	1	1	1	0	0	feces
190	2	1	1	0	0	feces
191	2	1	1	0	0	feces
192	2	1	1	0	0	feces
193	1	1	1	0	0	feces
194	2	1	1	0	0	feces

195	2	1	1	0	0	feces
196	2	1	1	0	0	feces
197	1	1	1	0	0	feces
198	2	1	1	0	0	feces
199	2	1	1	0	0	feces
200	2	1	1	0	0	feces
201	1	2	1	1	0	liver
202	2	2	1	1	0	liver
203	2	2	1	1	0	liver
204	2	2	1	1	0	liver
205	1	2	1	0	0	liver
206	2	2	1	0	0	liver
207	2	2	1	0	0	liver
208	2	2	1	0	0	liver
209	1	2	1	0	0	liver
210	2	2	1	0	0	liver
211	2	2	1	0	0	liver
212	2	2	1	0	0	liver
213	1	2	1	0	0	liver
214	2	2	1	0	0	liver
215	2	2	1	0	0	liver
216	2	2	1	0	0	liver
217	1	2	1	0	0	liver
218	2	2	1	0	0	liver
219	2	2	1	0	0	liver
220	2	2	1	0	0	liver
221	1	2	1	0	0	liver
222	2	2	1	0	0	liver
223	2	2	1	0	0	liver
224	2	2	1	0	0	liver
225	1	2	1	0	0	liver
226	2	2	1	0	0	liver
227	2	2	1	0	0	liver
228	2	2	1	0	0	liver
229	1	2	1	0	0	liver
230	2	2	1	0	0	liver
231	2	2	1	0	0	liver
232	2	2	1	0	0	liver
233	1	2	1	0	0	liver

234	2	2	1	0	0	liver
235	2	2	1	0	0	liver
236	2	2	1	0	0	liver
237	1	2	1	0	0	liver
238	2	2	1	0	0	liver
239	2	2	1	0	0	liver
240	2	2	1	0	0	liver
241	1	2	1	0	0	liver
242	2	2	1	0	0	liver
243	2	2	1	0	0	liver
244	2	2	1	0	0	liver
245	1	2	1	0	0	liver
246	2	2	1	0	0	liver
247	2	2	1	0	0	liver
248	2	2	1	0	0	liver
249	1	2	1	0	0	liver
250	2	2	1	0	0	liver
251	2	2	1	0	0	liver
252	2	2	1	0	0	liver
253	1	2	1	0	0	liver
254	2	2	1	0	0	liver
255	2	2	1	0	0	liver
256	2	2	1	0	0	liver
257	1	2	1	0	0	liver
258	2	2	1	0	0	liver
259	2	2	1	0	0	liver
260	2	2	1	0	0	liver

*Sex: 1-male, 2-female,
Feeding: 1-mill offal, 0-sheep offal
HEV: 1-positive, 0-negative,
Foreign introduction: 1-yes, 0-no.

Supplementary Table S6.
Reference Information of Research Sheep

№	Sex	Age	Location	HEV morbidity	sample
1	1	2	Bayanchandmani	1	feces
2	1	2	Bayanchandmani	1	feces
3	2	2	Batsumber	1	feces
4	2	2	Batsumber	1	feces
5	1	2	Bayanchandmani	0	feces
6	1	2	Bayanchandmani	0	feces
7	2	2	Bayanchandmani	0	feces
8	2	2	Bayanchandmani	0	feces
9	1	2	Bayanchandmani	0	feces
10	1	2	Bayanchandmani	0	feces
11	2	2	Bayanchandmani	0	feces
12	2	2	Bayanchandmani	0	feces
13	1	2	Bayanchandmani	0	feces
14	1	2	Bayanchandmani	0	feces
15	2	2	Bayanchandmani	0	feces
16	2	2	Bayanchandmani	0	feces
17	1	2	Bayanchandmani	0	feces
18	1	2	Bayanchandmani	0	feces
19	2	2	Bayanchandmani	0	feces
20	2	2	Bayanchandmani	0	feces
21	1	2	Bayanchandmani	0	feces
22	1	2	Bayanchandmani	0	feces
23	2	2	Bayanchandmani	0	feces
24	2	2	Bayanchandmani	0	feces
25	1	2	Bayanchandmani	0	feces
26	1	2	Bayanchandmani	0	feces
27	2	2	Bayanchandmani	0	feces
28	2	2	Bayanchandmani	0	feces
29	1	2	Bayanchandmani	0	feces
30	1	2	Bayanchandmani	0	feces
31	2	2	Bayanchandmani	0	feces
32	2	2	Bayanchandmani	0	feces
33	1	2	Bayanchandmani	0	feces
34	1	2	Bayanchandmani	0	feces
35	2	2	Bayanchandmani	0	feces

36	2	2	Bayanchandmani	0	feces
37	1	2	Bayanchandmani	0	feces
38	1	2	Bayanchandmani	0	feces
39	2	2	Bayanchandmani	0	feces
40	2	2	Bayanchandmani	0	feces
41	1	2	Bayanchandmani	0	feces
42	1	2	Bayanchandmani	0	feces
43	2	2	Bayanchandmani	0	feces
44	2	2	Bayanchandmani	0	feces
45	1	2	Bayanchandmani	0	feces
46	1	2	Bayanchandmani	0	feces
47	2	2	Bayanchandmani	0	feces
48	2	2	Bayanchandmani	0	feces
49	1	2	Bayanchandmani	0	feces
50	1	2	Bayanchandmani	0	feces
51	2	2	Bayanchandmani	0	feces
52	2	2	Bayanchandmani	0	feces
53	1	2	Batsumber	0	feces
54	1	2	Batsumber	0	feces
55	2	2	Batsumber	0	feces
56	2	2	Batsumber	0	feces
57	1	2	Batsumber	0	feces
58	1	2	Batsumber	0	feces
59	2	2	Batsumber	0	feces
60	2	2	Batsumber	0	feces
61	1	2	Batsumber	0	feces
62	1	2	Batsumber	0	feces
63	2	2	Batsumber	0	feces
64	2	2	Batsumber	0	feces
65	1	2	Batsumber	0	feces
66	1	2	Batsumber	0	feces
67	2	2	Batsumber	0	feces
68	2	2	Batsumber	0	feces
69	1	2	Batsumber	0	feces
70	1	2	Batsumber	0	feces
71	2	2	Batsumber	0	feces
72	2	2	Batsumber	0	feces
73	1	2	Batsumber	0	feces
74	1	2	Batsumber	0	feces
75	2	2	Batsumber	0	feces

76	2	2	Batsumber	0	feces
77	1	2	Batsumber	0	feces
78	1	2	Batsumber	0	feces
79	2	2	Batsumber	0	feces
80	2	2	Batsumber	0	feces
81	1	2	Batsumber	0	feces
82	1	2	Batsumber	0	feces
83	2	2	Batsumber	0	feces
84	2	2	Batsumber	0	feces
85	1	2	Batsumber	0	feces
86	1	2	Batsumber	0	feces
87	2	2	Batsumber	0	feces
88	2	2	Batsumber	0	feces
89	1	2	Batsumber	0	feces
90	1	2	Batsumber	0	feces
91	2	2	Batsumber	0	feces
92	2	2	Batsumber	0	feces
93	1	2	Batsumber	0	feces
94	1	2	Batsumber	0	feces
95	2	2	Batsumber	0	feces
96	2	2	Batsumber	0	feces
97	1	2	Batsumber	0	feces
98	1	2	Batsumber	0	feces
99	2	2	Batsumber	0	feces
100	2	2	Batsumber	0	feces
101	1	1	Zaamar	0	feces
102	1	1	Zaamar	0	feces
103	2	1	Zaamar	0	feces
104	2	1	Zaamar	0	feces
105	1	1	Zaamar	0	feces
106	1	1	Zaamar	0	feces
107	2	1	Zaamar	0	feces
108	2	1	Zaamar	0	feces
109	1	1	Zaamar	0	feces
110	1	1	Zaamar	0	feces
111	2	1	Zaamar	0	feces
112	2	1	Zaamar	0	feces
113	1	1	Zaamar	0	feces
114	1	1	Zaamar	0	feces

115	2	1	Zaamar	0	feces
116	2	1	Zaamar	0	feces
117	1	1	Zaamar	0	feces
118	1	1	Zaamar	0	feces
119	2	1	Zaamar	0	feces
120	2	1	Zaamar	0	feces
121	1	1	Zaamar	0	feces
122	1	1	Zaamar	0	feces
123	2	1	Zaamar	0	feces
124	2	1	Zaamar	0	feces
125	1	1	Zaamar	0	feces
126	1	1	Zaamar	0	feces
127	2	1	Zaamar	0	feces
128	2	1	Zaamar	0	feces
129	1	1	Zaamar	0	feces
130	1	1	Zaamar	0	feces
131	2	1	Zaamar	0	feces
132	2	1	Zaamar	0	feces
133	1	1	Zaamar	0	feces
134	1	1	Zaamar	0	feces
135	2	1	Zaamar	0	feces
136	2	1	Zaamar	0	feces
137	1	1	Zaamar	0	feces
138	1	1	Zaamar	0	feces
139	2	1	Zaamar	0	feces
140	2	1	Zaamar	0	feces
141	1	1	Zaamar	0	feces
142	1	1	Zaamar	0	feces
143	2	1	Zaamar	0	feces
144	2	1	Zaamar	0	feces
145	1	1	Zaamar	0	feces
146	1	1	Zaamar	0	feces
147	2	1	Zaamar	0	feces
148	2	1	Zaamar	0	feces
149	1	1	Zaamar	0	feces
150	1	1	Zaamar	0	feces
151	2	1	Undurshireet	0	feces
152	2	1	Undurshireet	0	feces
153	1	1	Undurshireet	0	feces
154	1	1	Undurshireet	0	feces

155	2	1	Undurshireet	0	feces
156	2	1	Undurshireet	0	feces
157	1	1	Undurshireet	0	feces
158	1	1	Undurshireet	0	feces
159	2	1	Undurshireet	0	feces
160	2	1	Undurshireet	0	feces
161	1	1	Undurshireet	0	feces
162	1	1	Undurshireet	0	feces
163	2	1	Undurshireet	0	feces
164	2	1	Undurshireet	0	feces
165	1	1	Undurshireet	0	feces
166	1	1	Undurshireet	0	feces
167	2	1	Undurshireet	0	feces
168	2	1	Undurshireet	0	feces
169	1	1	Undurshireet	0	feces
170	1	1	Undurshireet	0	feces
171	2	1	Undurshireet	0	feces
172	2	1	Undurshireet	0	feces
173	1	1	Undurshireet	0	feces
174	1	1	Undurshireet	0	feces
175	2	1	Undurshireet	0	feces
176	2	1	Undurshireet	0	feces
177	1	1	Undurshireet	0	feces
178	1	1	Undurshireet	0	feces
179	2	1	Undurshireet	0	feces
180	2	1	Undurshireet	0	feces
181	1	1	Undurshireet	0	feces
182	1	1	Undurshireet	0	feces
183	2	1	Undurshireet	0	feces
184	2	1	Undurshireet	0	feces
185	1	1	Undurshireet	0	feces
186	1	1	Undurshireet	0	feces
187	2	1	Undurshireet	0	feces
188	2	1	Undurshireet	0	feces
189	1	1	Undurshireet	0	feces
190	1	1	Undurshireet	0	feces
191	2	1	Undurshireet	0	feces
192	2	1	Undurshireet	0	feces
193	1	1	Undurshireet	0	feces

194	1	1	Undurshireet	0	feces
195	2	1	Undurshireet	0	feces
196	2	1	Undurshireet	0	feces
197	1	1	Undurshireet	0	feces
198	1	1	Undurshireet	0	feces
199	2	1	Undurshireet	0	feces
200	2	1	Undurshireet	0	feces
201	1	2	Bayanchandmani	1	liver
202	1	2	Bayanchandmani	1	liver
203	2	2	Bayanchandmani	1	liver
204	2	2	Bayanchandmani	0	liver
205	1	2	Bayanchandmani	0	liver
206	1	2	Bayanchandmani	0	liver
207	2	2	Bayanchandmani	0	liver
208	2	2	Bayanchandmani	0	liver
209	1	2	Bayanchandmani	0	liver
210	1	2	Bayanchandmani	0	liver
211	2	2	Bayanchandmani	0	liver
212	2	2	Bayanchandmani	0	liver
213	1	2	Bayanchandmani	0	liver
214	1	2	Bayanchandmani	0	liver
215	2	2	Bayanchandmani	0	liver
216	2	2	Batsumber	0	liver
217	1	2	Batsumber	0	liver
218	1	2	Batsumber	0	liver
219	2	2	Batsumber	0	liver
220	2	2	Batsumber	0	liver
221	1	2	Batsumber	0	liver
222	1	2	Batsumber	0	liver
223	2	2	Batsumber	0	liver
224	2	2	Batsumber	0	liver
225	1	2	Batsumber	0	liver
226	1	2	Batsumber	0	liver
227	2	2	Batsumber	0	liver
228	2	2	Batsumber	0	liver
229	1	2	Batsumber	0	liver
230	1	2	Batsumber	0	liver
231	2	2	Zaamar	0	liver
232	2	2	Zaamar	0	liver
233	1	2	Zaamar	0	liver

234	1	2	Zaamar	0	liver
235	2	2	Zaamar	0	liver
236	2	2	Zaamar	0	liver
237	1	2	Zaamar	0	liver
238	1	2	Zaamar	0	liver
239	2	2	Zaamar	0	liver
240	2	2	Zaamar	0	liver
241	1	2	Zaamar	0	liver
242	1	2	Zaamar	0	liver
243	2	2	Zaamar	0	liver
244	2	2	Zaamar	0	liver
245	1	2	Zaamar	0	liver
246	1	2	Undurshireet	0	liver
247	2	2	Undurshireet	0	liver
248	2	2	Undurshireet	0	liver
249	1	2	Undurshireet	0	liver
250	1	2	Undurshireet	0	liver
251	2	2	Undurshireet	0	liver
252	2	2	Undurshireet	0	liver
253	1	2	Undurshireet	0	liver
254	1	2	Undurshireet	0	liver
255	2	2	Undurshireet	0	liver
256	2	2	Undurshireet	0	liver
257	1	2	Undurshireet	0	liver
258	1	2	Undurshireet	0	liver
259	2	2	Undurshireet	0	liver
260	2	2	Undurshireet	0	liver

Sex 1-male, 2-female

Age 1-less than 1 year, 2-Over 1 year

HEV 0-negative, 1-HEV positive
